# A Deep Learning-Based Approach for Explainable Microsatellite Instability Detection in Gastrointestinal Malignancies

**DOI:** 10.3390/jimaging11110398

**Published:** 2025-11-07

**Authors:** Ludovica Ciardiello, Patrizia Agnello, Marta Petyx, Fabio Martinelli, Mario Cesarelli, Antonella Santone, Francesco Mercaldo

**Affiliations:** 1 Department of Medicine and Health Sciences “Vincenzo Tiberio”, University of Molise, 86100 Campobasso, Italy; antonella.santone@unimol.it; 2 Istituto Nazionale per l’Assicurazione contro gli Infortuni sul Lavoro, 00144 Rome, Italy; p.agnello@inail.it (P.A.); m.petyx@inail.it (M.P.); 3 Institute for High Performance Computing and Networking, National Research Council of Italy (CNR), 87036 Rende, Italy; fabio.martinelli@icar.cnr.it; 4 Department of Engineering, University of Sannio, 82100 Benevento, Italy; mcesarelli@unisannio.it

**Keywords:** microsatellite instability, deep learning, convolutional neural network, explainability, Class Activation Mapping

## Abstract

Microsatellite instability represents a key biomarker in gastrointestinal cancers with significant diagnostic and therapeutic implications. Traditional molecular assays for microsatellite instability detection, while effective, are costly, time-consuming, and require specialized infrastructure. In this paper we propose an explainable deep learning-based method for microsatellite instability detection starting from the analysis of histopathological images. We consider a set of convolutional neural network architectures i.e., MobileNet, Inception, VGG16, VGG19, and a Vision Transformer model, and we propose a way to provide a kind of clinical explainability behind the model prediction through (three) Class Activation Mapping techniques. With the aim to further strengthen trustworthiness in predictions, we introduce a set of robustness metrics aimed to quantify the consistency of highlighted discriminative regions across different Class Activation Mapping methods. Experimental results on a real-world dataset demonstrate that VGG16 and VGG19 models achieve the best performance in terms of accuracy; in particular, the VGG16 model obtains an accuracy of 0.926, while the VGG19 one reaches an accuracy equal to 0.917. Furthermore, Class Activation Mapping techniques confirmed that the developed models consistently focus on similar tissue regions, while robustness analysis highlighted high agreement between different Class Activation Mapping techniques. These results indicate that the proposed method not only achieves interesting predictive accuracy but also provides explainable predictions, with the aim to boost the integration of deep learning into real-world clinical practice.

## 1. Introduction

Microsatellite instability (MSI) is a form of genetic hypermutability that arises from impaired DNA mismatch repair. It is a well-established biomarker in gastrointestinal cancers, particularly colorectal and gastric carcinoma, and has important prognostic and therapeutic implications [1,2,3,4]. From a pathological point of view, MSI tumors typically present with glandular heterogeneity, poor differentiation, and dense intratumoral lymphocytic infiltration, reflecting an active immune response driven by the accumulation of neoantigens [1,2]. Conversely, microsatellite stable (MSS) tumors tend to be more morphologically homogeneous, better differentiated, and characterized by limited lymphocytic infiltration [3,4]. Reliable identification of MSI status is therefore critical, both for guiding immunotherapy decisions and for improving diagnostic accuracy in routine clinical practice.

Traditionally, MSI status is assessed using molecular assays such as polymerase chain reaction (PCR)-based testing or immunohistochemistry (IHC) to evaluate the expression of mismatch repair (MMR) proteins. While effective, these approaches can be costly, time-consuming, and dependent on the availability of specialized laboratory infrastructure. The growing availability of digital pathology and whole-slide imaging has opened new opportunities to explore computational pathology approaches that leverage machine learning and deep learning techniques for automated MSI classification directly from histopathological images [5]. Such methods promise scalable and cost-effective solutions that could complement or even replace conventional assays in certain clinical scenarios.

Convolutional neural networks (CNNs) have demonstrated remarkable performance in image-based classification due to their ability to learn hierarchical feature representations from raw input data, also in biomedical contexts [6,7,8,9]. As a matter of fact, with regard to gastrointestinal cancers, CNNs have been successfully applied to tasks such as tumor detection, grading, and molecular phenotype prediction [5]. However, despite their predictive ability, CNN-based models are not widely employed in clinical practice for their limited explainability, which poses critical challenges for real-world clinical adoption. As a matter of fact, explainability is essential to ensure that the regions highlighted as predictive truly correspond to biologically meaningful structures, thereby fostering trust among pathologists and clinicians.

Starting from these considerations, in this paper we propose a method for MSI detection in gastrointestinal cancer histology images through CNNs, by exploiting a set of models. Furthermore, to incorporate prediction explainability into the predictions of the model, we employ a set of Class Activation Mapping (CAM)-based techniques, to visualize how different image regions contribute to the model’s decisions. We also assess model stability, by introducing a set of metrics, which quantify the consistency across different visualization methods. This allows us not only to evaluate the prediction accuracy between MSI and MSS but also to provide a kind of stability of explanations across different CAM methods, thereby supporting the integration of deep learning into real-world pathology workflows.

## 2. Materials and Methods

In this section we introduce the proposed method, aimed to detect the presence of MSI directly from histology in gastrointestinal cancer by providing explainability behind the model prediction.

In detail the proposed method is aimed at providing a binary classification model to assign, to a tissue image related to gastrointestinal cancer, the MSS, or the MSI label.

### 2.1. Dataset

To evaluate the proposed method, we consider a dataset freely available for research purposes, published by Jakob Nikolas Kather [5] in February 2019. The exploited dataset is composed of 411,890 different images, obtained from histological samples of patients with colorectal and gastric cancer, belonging to the TCGA (The Cancer Genome Atlas) cohort. The images were derived from FFPE diagnostic slides (formalin-fixed, paraffin-embedded https://andrewjanowczyk.com/download-tcga-digital-pathology-images-ffpe/ (accessed on 6 October 2025) and subsequently divided into patches of 224 × 224 pixels, with a resolution of 0.5 µm/px.

These patches belong to two distinct groups:

- **MSS**: tumors without significant mutations in microsatellite regions.
- **MSI**: tumors with a high frequency of mutations in microsatellites.

**Figure 1:**
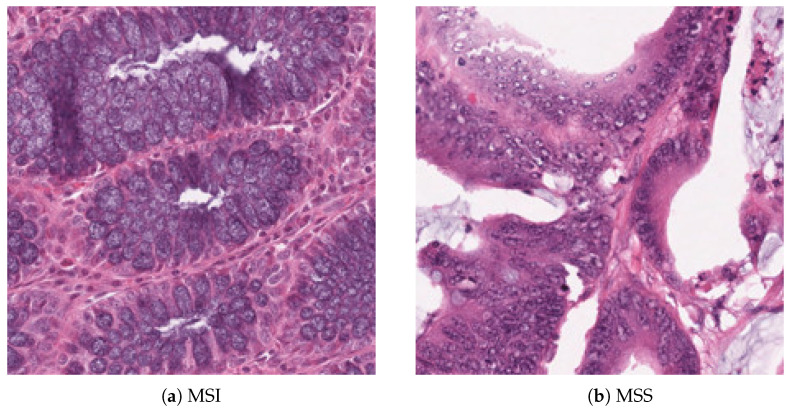
Comparison between MSI and MSS tissues.

In gastrointestinal carcinoma with MSI, as observable in the first image shown in Figure 1a, the glandular architecture appears markedly heterogeneous, with glands varying in shape and size. This morphological heterogeneity is typical of MSI carcinomas, in which a deficiency in the DNA mismatch repair (MMR) system leads to a high mutation rate, promoting intratumoral structural polymorphism. From a cytological perspective, the cells show poor differentiation, with large and hyperchromatic nuclei [1]. A distinctive feature is the presence of a dense intratumoral lymphocytic infiltrate, distributed both in the peri-glandular stroma and at the intraepithelial level. This finding is consistent with the MSI phenotype, which is known to be associated with a strong immune response driven by the elevated production of tumor neoantigens [2,3].

Conversely, in MSS tumors, as shown in the second image in Figure 1b, the architecture is more homogeneous and regular, with glands of relatively uniform shape and arrangement, displaying a predominantly glandular pattern. The cells are generally better differentiated, with nuclei more uniform in size and shape. The lymphocytic infiltrate is less abundant and mainly confined to the stroma, with scarce intraepithelial lymphocytes, characteristic of a more attenuated immune response typical of this phenotype [4].

### 2.2. Dataset Preprocessing

The images underwent the following preprocessing steps performed by the original dataset contributors:

- Automated Tumor Area Identification: Regions containing tumor tissue were automatically detected, excluding non-relevant areas such as healthy tissue, stroma, and artifacts.
- Image Size Standardization: All patches were resized to 224 × 224 pixels with a fixed resolution of 0.5 μm per pixel.
- Color Normalization Using Macenko Method: To reduce color variations caused by differences in histological slide staining, the color normalization technique proposed by Macenko et al. (2009) [10] was applied.
- Patient Classification: Each sample was labeled as MSS or MSIMUT based on the tumor’s genetic and molecular characteristics.

A total of 192,310 histological images were selected. The dataset, https://www.kaggle.com/datasets/joangibert/tcga_coad_msi_mss_jpg, accessed on 6 October 2025, used in this study was obtained from a public repository on Kaggle and was randomly divided into training, validation, and test sets according to an 80%—10%—10% split. Class representation was preserved across all subsets.

The **training set** consists of 153,849 images in total, of which 60,031 belong to the MSI class and 93,818 to the MSS class.

The **validation set** includes 19,230 images in total, with 7503 labeled as MSI and 11,727 as MSS.

The **test set** is composed of 19,230 images, including 7502 from the MSIMUT class and 11,728 from the MSS class.

### 2.3. Experimental Settings

We consider a set of experiments, wherein in each experiment the hyperparameters used for model training are varying. Below we describe the set of hyperparameters we consider:

- **Number of epochs**: indicates how many times the algorithm processes the entire dataset during training.
- **Batch size**: represents the number of samples processed simultaneously before the network weights are updated.
- **Image size**: corresponds to the resolution of the input images provided to the network.
- **Learning rate**: defines the speed at which the model’s weights are updated during optimization.

Several neural network models were trained using the dataset under study. Table 1 summarizes the main hyperparameters that were varied during training.

As shown in Table 1, the experiments were conducted with a fixed learning rate of $1\times 10^{-5}$.

Two image resolutions were used: 224 × 224 × 3 (original) and 120 × 120 × 3 (downscaled), to assess the impact of image resolution on both learning accuracy and training time.

The number of epochs ranged from 6 to 30, and batch sizes of 16 and 32 were tested to better compare model performance.

Moreover, considering that the provided classification task is binary, the decision threshold is set to 50%.

Training times varied depending on the configuration, ranging from 1 h and 56 min to 8 h and 23 min. All experiments were performed on an NVIDIA GeForce RTX 4070 GPU (NVIDIA; Santa Clara, CA, USA) with 8 GB of memory, supported by an Intel Core i7-14700HX CPU (Intel; Santa Clara, CA, USA) with 20 cores and 28 threads at 2.1 GHz and 32 GB of RAM.

### 2.4. Model Training and Testing

**Figure 2:**
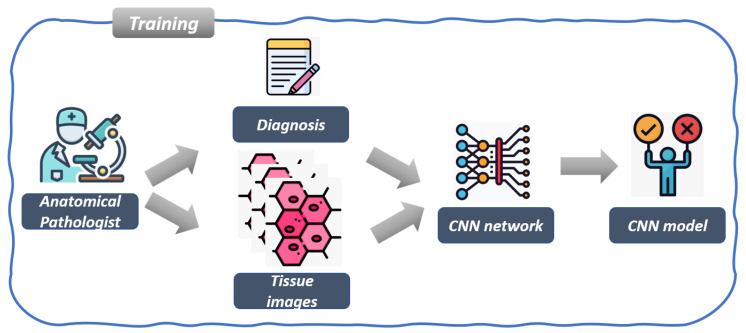
The training step of the proposed method for MSI detection.

**Figure 3:**
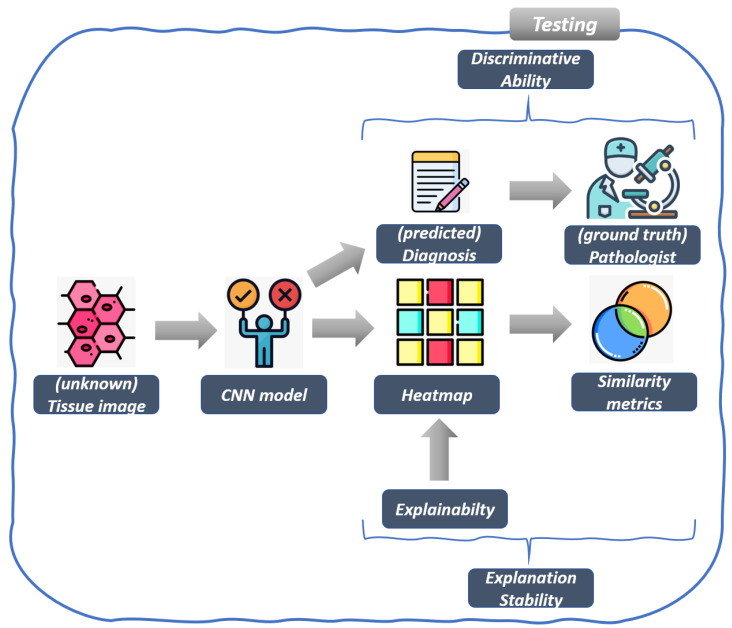
The testing step of the proposed method for MSI detection.

The workflow of the proposed method is shown in Figure 2 and Figure 3 in detail. Figure 2 is related to the model training, while Figure 3 shows the workflow of the model testing, by detailing the explainability and robustness steps.

The first step shown in Figure 2 is related to the acquisition of histopathological images by anatomical pathologists, who examine tissue samples using microscopy. As a matter of fact, experts are responsible not only for capturing high-resolution tissue images but also for annotating each tissue image with a clinical diagnosis, indicating whether the tissue is related to the MSI or the MSS class. The aim of this first step results in a labeled dataset that is exploited for model training.

As a matter of fact, once the dataset of annotated tissue images is acquired, for training we consider a CNN model. Each tissue image, along with its related label, is fed into each CNN architecture during the training phase. The aim of the CNN network is to automatically learn meaningful hierarchical features from the tissue morphology that can differentiate tissues related to the MSI class from the MSS one. We exploit several CNN architectures to determine the most effective one for the classification task detail. We resort to the following CNNs: MobileNet [11], Inception [12], VGG16 and VGG19 [13].

MobileNet is a CNN optimized for mobile and embedded vision tasks, achieving efficiency by replacing standard convolutions with depthwise separable convolutions. This reduces computational cost from $D_{k}^{2}MN$ multiplications in a standard convolution to $D_{k}^{2}M+MN$. Two hyperparameters, the width multiplier α and resolution multiplier ρ, allow explicit trade-offs between accuracy, latency, and memory, making MobileNet well-suited for low-power devices.

The Inception architecture improves representational power through multi-branch modules that apply convolutions of different kernel sizes (1×1, 3×3, 5×5) and pooling in parallel, concatenating their outputs, while dimensionality reduction with 1×1 convolutions controls complexity.

VGG16, introduced by the Visual Geometry Group, follows a simple and uniform design of stacked 3×3 convolutions with max-pooling, comprising 13 convolutional and 3 fully connected layers (about 138M parameters). VGG19 extends this with three additional convolutional layers, increasing representational power at the cost of higher computation (>143 M parameters). Despite their size, both models became widely adopted for transfer learning and as backbones in detection and segmentation tasks.

We consider different CNNs, considering that each network exhibits different characteristics; for instance the MobileNet model emphasizes efficiency through depthwise separable convolutions and tunable hyperparameters, making it optimal for edge devices. Inception introduces a multi-scale processing paradigm within each layer, combining convolutional kernels of different sizes for richer feature representation while controlling computational cost through dimensionality reduction. VGG16 and VGG19 prioritize architectural uniformity, relying on deep stacks of small convolutional filters to achieve high accuracy, albeit at significant memory and computation requirements. In a nutshell, MobileNet is related to resource-constrained scenarios, Inception for scalable yet efficient feature extraction, and VGG networks for simplicity and transfer learning utility.

The output of the training process is represented by a CNN-based classification model capable of assigning the MSI or the MSS class to unseen tissue images.

Moreover, we also consider a model based on the Vision Transformer (ViT) architecture i.e., a transformer-based model tailored for image classification tasks. Specifically, we resort to the pretrained *WinKawaks/vit-tiny-patch16-224* model, accessible via the Hugging Face transformers library https://huggingface.co/WinKawaks/vit-tiny-patch16-224, accessed on 6 October 2025.

The second step of the proposed method is related to the testing phase of the model built in the training step shown in Figure 2 and it is illustrated in Figure 3: in this step, from an *unseen tissue image*, the trained CNN model outputs a label (i.e., a *diagnosis*), predicting whether the tissue is related to the MSI or the MSS class.

### 2.5. Explainability and Explanations Stability

The proposed method also takes into account explainability, aimed at understanding which areas of the tissue image under analysis are symptomatic of a certain prediction, for this task, we generate a *heatmap* aimed at highlighting the most discriminative regions contributing to the classification decision (i.e., to the diagnosis).

To generate heatmaps we consider several CAM techniques: each involved technique estimates the contribution of different spatial regions to the final prediction, thereby providing visual insight into the model’s internal reasoning. The idea of this step is to provide the explainability of the model, which is crucial for the adoption of deep learning in real-world clinical practice. In particular, in this work we resort to three different CAM techniques, i.e., Gradient-weighted Class Activation Mapping (Grad-CAM) [14,15], Grad-CAM++ [16] and Score-CAM-Fast [17].

Grad-CAM generates class-discriminative heatmaps by computing the gradient of the target class score with respect to the feature maps of a chosen convolutional layer. These gradients are globally average-pooled to obtain importance weights, which are then linearly combined with the feature maps.

Grad-CAM++ builds upon this approach by introducing pixel-wise (spatial) weighting of gradients, allowing it to better capture multiple object instances and fine-grained details. It leverages higher-order derivatives to calculate per-pixel contribution factors, which are used to refine the feature-map weights.

In contrast, Score-CAM is a gradient-free method. It evaluates the contribution of each upsampled feature map to the class score by masking the input image with the feature map and performing forward passes, thereby estimating the influence of each region on the prediction.

Although Grad-CAM, Grad-CAM++, and Score-CAM Fast share are the aim of providing class-discriminative visual explanations for convolutional neural networks, they differ in computational design and the fidelity of localization. Grad-CAM is computationally efficient, relying only on first-order gradients and a single backward pass, but it assumes that the importance of each feature map can be represented by a single scalar weight, which can blur localization when objects are small or appear multiple times. Grad-CAM++ addresses this limitation by computing pixel-wise weights derived from higher-order derivatives, enabling sharper and more accurate heatmaps that capture multiple object instances, albeit with additional computational cost due to second- and third-order differentiation. In contrast, Score-CAM eliminates gradient computations entirely and evaluates the contribution of each feature map through forward passes, producing explanations that are often less noisy and less sensitive to gradient saturation. However, its baseline implementation is computationally expensive because it requires a forward pass per feature map; Score-CAM (Fast) mitigates this by approximations, sampling strategies, or map clustering, balancing explainability with practical scalability.

As mentioned above, each CAM technique uses a specific algorithm to highlight the areas that were found to be crucial for a prediction, thus providing an explanation behind the model’s prediction. Therefore, given a tissue image as input to a model, all CAMs should highlight the same area of interest. To evaluate this aspect, and therefore to understand whether the trained model consistently focuses on similar regions of tissue, we introduce a set of metrics aimed at evaluating the model’s robustness from this point of view. The basic idea is to quantify how much the CAMs agree on the highlighted areas related to the diagnosis.

Thus, we compute several indices; the first one is the *Structural Similarity Index Measure (SSIM)* [18] between heatmaps generated by different CAM techniques applied to the same tissue image and CNN model. SSIM quantifies the similarity between two images in terms of luminance, contrast, and structure. The SSIM between two heatmaps *x* and *y* is defined as follows:

(1) $$\mathrm{SSIM}\left(x,y\right)=\frac{\left(2\mu_{x}\mu_{y}+C_{1}\right)\left(2\sigma_{xy}+C_{2}\right)}{\left(\mu_{x}^{2}+\mu_{y}^{2}+C_{1}\right)\left(\sigma_{x}^{2}+\sigma_{y}^{2}+C_{2}\right)},$$

where $\mu_{x}$ and $\mu_{y}$ are the mean intensities, $\sigma_{x}^{2}$ and $\sigma_{y}^{2}$ are the variances, and $\sigma_{xy}$ is the covariance of images *x* and *y*. The constants $C_{1}$ and $C_{2}$ are used to stabilize the division when the denominators are small.

To obtain an overall robustness score, we compute the SSIM between all pairwise combinations of heatmaps for a given image and then *average the SSIM scores across the entire test set*. The resulting value lies in the range [0,1], where a score close to 1 indicates strong agreement among different CAM techniques. This consistency suggests that the model is focusing on similar regions across different explainability methods, thus enhancing confidence in its decision-making process.

In addition to SSIM, we also consider other indices i.e., PSNR, SAM, ERGAS, and VIF, with their related definitions written as follows:

(2) $$\mathrm{MSE}=\frac{1}{MN}\sum_{i=1}^{M}\sum_{j=1}^{N}{\left[I\left(i,j\right)-K\left(i,j\right)\right]}^{2}$$

(3) $$\mathrm{PSNR}=10\cdot \log_{10}\left(\frac{L^{2}}{\mathrm{MSE}}\right)$$

where *I* and *K* denote the reference and test images, respectively, and *L* is the maximum possible pixel value (e.g., 255 for 8-bit images). Higher PSNR indicates better image reconstruction quality.

The Peak Signal-to-Noise Ratio (PSNR) quantifies the ratio between the maximum possible signal power and the noise affecting image fidelity. It is derived from the Mean Squared Error (MSE):

The Spectral Angle Mapper (SAM) measures the spectral similarity between two image vectors by computing the mean spectral angle between corresponding pixels:

(4) $$\mathrm{SAM}\left(I,K\right)=\frac{1}{MN}\sum_{i=1}^{M}\sum_{j=1}^{N}\cos^{-1}\left(\frac{\langle I_{ij},K_{ij}\rangle }{{∥I_{ij}∥}_{2}\;{∥K_{ij}∥}_{2}}\right)$$

where $I_{ij}$ and $K_{ij}$ are the pixel vectors at position (i,j), 〈·,·〉 denotes the dot product, and ${∥\cdot ∥}_{2}$ is the Euclidean norm. Smaller SAM values indicate higher spectral similarity.

Erreur Relative Globale Adimensionnelle de Synthèse (ERGAS) quantifies the overall relative error between corresponding bands of two multispectral images:

(5) $$\mathrm{ERGAS}=100\times \frac{h}{l}\sqrt{\frac{1}{N_{b}}\sum_{b=1}^{N_{b}}{\left(\frac{{\mathrm{RMSE}}_{b}}{\mu_{b}}\right)}^{2}}$$

where $N_{b}$ is the number of spectral bands, ${\mathrm{RMSE}}_{b}$ and $\mu_{b}$ are the root mean square error and mean of the reference image in band *b*, respectively, and h/l represents the ratio of high- to low-resolution pixel sizes. Lower ERGAS values correspond to better image quality.

The Visual Information Fidelity (VIF) measures the amount of information preserved in the distorted image relative to the reference image, based on natural scene statistics and mutual information in the wavelet domain:

(6) $$\mathrm{VIF}=\frac{\sum_{j}\sum_{i\in \mathrm{subband}\;j}\log_{2}\left(1+\frac{g_{j}^{2}\sigma_{x_{j},i}^{2}}{\sigma_{v_{j},i}^{2}+\sigma_{n_{j},i}^{2}}\right)}{\sum_{j}\sum_{i\in \mathrm{subband}\;j}\log_{2}\left(1+\frac{\sigma_{x_{j},i}^{2}}{\sigma_{n_{j},i}^{2}}\right)}$$

where $\sigma_{x_{j},i}^{2}$ is the variance of the reference image signal, $\sigma_{n_{j},i}^{2}$ is the visual noise variance, $\sigma_{v_{j},i}^{2}$ is the distortion noise variance, and $g_{j}$ is the gain factor in subband *j*. VIF values range between 0 and 1, with higher values indicating greater visual fidelity.

By combining prediction, explainability through heatmaps, and evaluation via these five indices, the proposed method aims to ensure an explainable diagnostic framework suitable for deployment in real-world clinical environments.

## 3. Results

In this section we present the experimental results we performed on a real-world dataset.

### 3.1. Experimental Results

Table 2 shows the results of the 11 experiments we conducted, evaluated through the metrics of loss, accuracy, precision, recall, F-Measure, and AUC. Overall, the models are able to discriminate between MSI and MSS images, with a maximum AUC of 0.9760, indicating an interesting ability to discriminate between the two classes. In particular, precision, recall, and F-Measure reached 0.9178, with an accuracy of 0.9178.

The best performances were obtained with VGG16 and VGG19. Experiment 6 in Table 2 with VGG16 achieved an accuracy of 0.926, with precision, recall, and F-Measure at 0.9260, a loss of 0.2685, and an AUC of 0.9730. Experiment 8 with VGG19 reached an accuracy of 0.9178, with precision, recall, and F-Measure at 0.9178, a loss of 0.2064, and an AUC of 0.9760. Notably, both experiments shared the same hyperparameters, as shown in Table 1, except for the number of epochs (12 for VGG16 and 11 for VGG19), yet still achieved excellent results.

Experiments with Inception (Exp 3 and 4) shown in Table 2 showed stable performance, although slightly lower than that of the VGG models.

The lowest performance was observed in Experiment 2 in Table 2 with MobileNet, where the loss was 1.1026 and all other metrics (accuracy, precision, recall, and F-Measure) were 0.5722, with an AUC of 0.5985. Interestingly, the same network with different hyperparameters in Experiment 1 achieved considerably better results, with a loss of 0.5242, accuracy, precision, recall, and F-Measure all at 0.8267, and an AUC of 0.9060, highlighting the model’s sensitivity to training configuration.

The ViT model (Experiment 12) achieved a moderate performance, with an accuracy of 0.6812 and an AUC of 0.6972. The relatively high loss value (0.9226) suggests that ViT may not have generalized as effectively as other architectures we considered in the experiment analysis if compared to CNN-based models.

**Figure 4:**
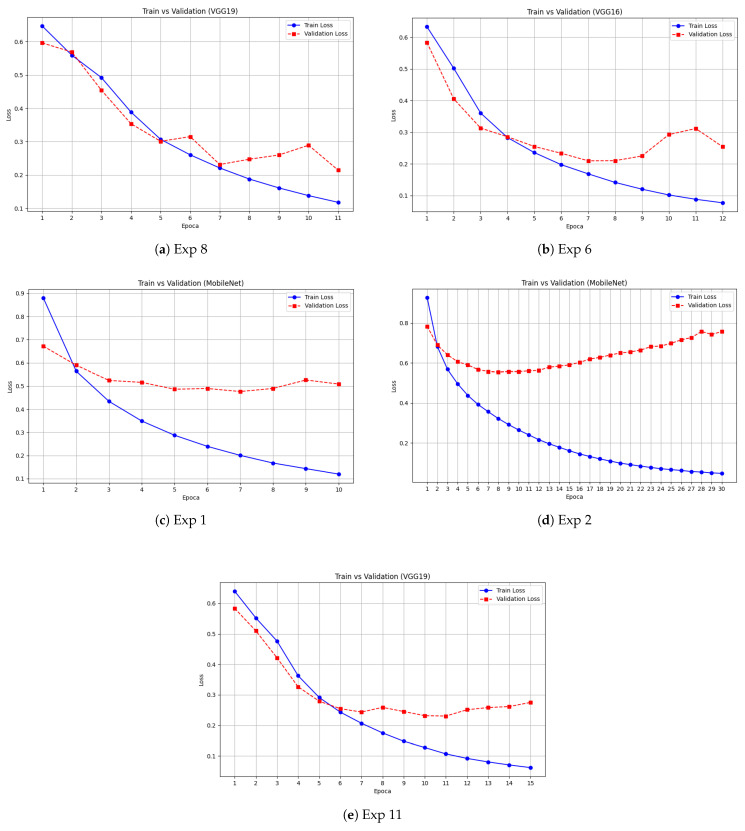
Training and validation loss trends for different experiments across VGG16, VGG19, and MobileNet.

Figure 4 show the plots related to the loss trends during training and validation. Experiment 6 in Figure 4b and experiment 8 Figure 4a, respectively corresponding to VGG16 and VGG19, shows a consistent trend between training and validation, in line with the test results reported in Table 2. In contrast, Experiment 11 Figure 4e shows that while the training loss reaches optimal values, the validation loss starts to diverge, indicating potential overfitting.

For MobileNet, the two experiments illustrate different behaviors: in Experiment 1 shown in Figure 4c, the validation loss remains relatively stable, whereas in Experiment 2 shown in Figure 4d, the present of overfitting is evident, with the model learning well during training but failing to generalize adequately to the validation set.

### 3.2. Explainability and Robustness Analysis

With regard to the explainability analysis, in the following we consider a set of examples obtained from the best-performing model, corresponding to Experiment 8 shown in Table 2. Each CAM technique we consider produces a heatmap aimed to provide a representation of the spatial importance of features: the most relevant areas are shown in yellow/green, while less significant regions appear in blue/purple.

In fact, for each analyzed example, explainability analysis is presented through three images:

- The original image, with the predicted class and associated probability;
- The CAM heatmap;
- The overlay of the heatmap on the original image, allowing a clear and immediate visual assessment of the regions critical for classification.

**Figure 5:**
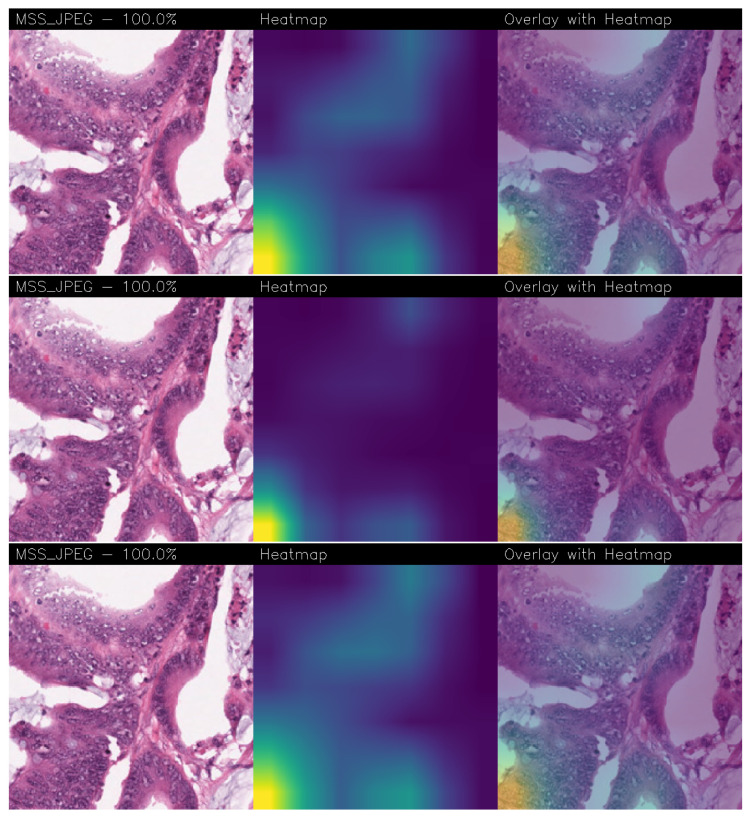
Grad-CAM (on the **top**), Grad-CAM++ (in the **middle**) and Score-CAM Fast (on the **bottom**) explainability related to a MSS tissue.

**Figure 6:**
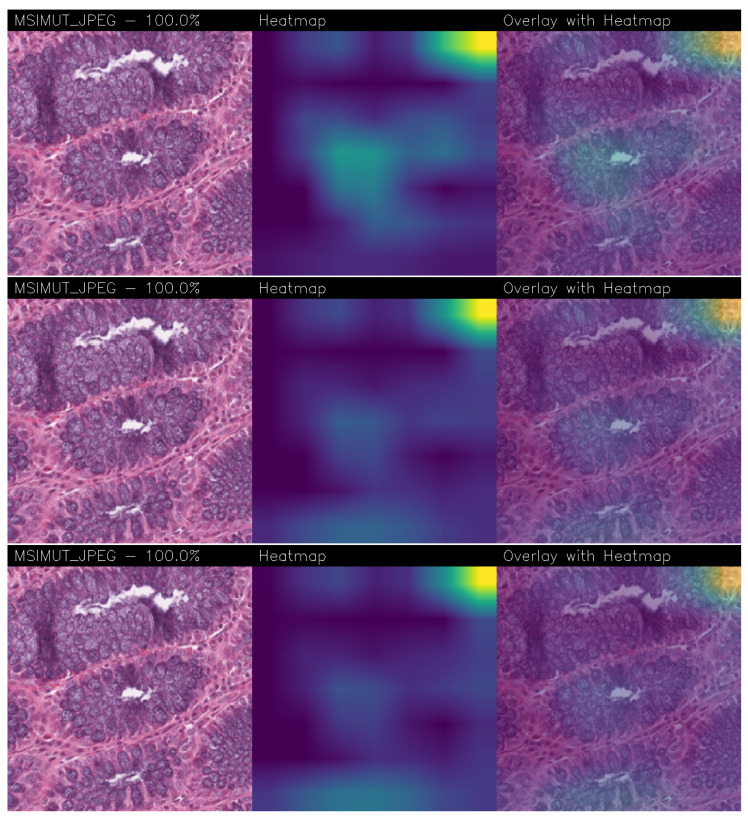
Grad-CAM (on the **top**), Grad-CAM++ (in the **middle**), and Score-CAM Fast (on the **bottom**) explainability related to a MSI tissue.

In the following we provide several examples of explainability provided by the proposed method: in particular, in Figure 5, the Grad-CAM, Grad-CAM++, and Score-CAM Fast explainability with an MSS image predicted as rightly belonging to the MSS class with a probability equal to 100% is shown, while in Figure 6, we depict an example of Grad-CAM, Grad-CAM++, and Score-CAM Fast explainability with an MSI image, also in this case predicted with a probability of 100% as MSI.

The visual similarities between the heatmaps generated by different CAM methods were evaluated using the SSIM index. The results, shown in Table 3, confirm that all comparisons exhibit values above 50%, thus exhibiting a good degree of agreement among the regions of interest identified by the different methods and thus providing robustness behind explainability.

As reported in Table 3, for the MSS class, the highest similarity is observed between Score-CAM Fast and Grad-CAM (SSIM = 0.93), while for the MSI class, the best agreement is between Grad-CAM++ and Grad-CAM (SSIM = 0.84). Considering both classes overall, the comparison between Score-CAM Fast and Grad-CAM yields the highest SSIM values, indicating the strongest overall concordance.

High SSIM values suggest that the models agree on the relevant regions of the images, focusing on areas that are significant for correctly distinguishing between the two classes. This confirms the ability of CAM methods to consistently highlight discriminative regions, thereby supporting explainability (and also robustness) of deep learning models.

In addition to the quantitative evaluation, the heatmaps generated by the different CAM techniques show a focus on similar regions of the images. This indicates an agreement between the methods in highlighting the same relevant areas. The SSIM index provides a numerical measure of this concordance, quantifying the similarity between the heatmaps produced by the different approaches. Overall, the visual inspection and the SSIM values consistently show that the different CAM techniques converge on comparable regions of interest.

Table 4 reports the quantitative comparison among the different CAM methods based on four image quality metrics: VIF, SAM, ERGAS, and PSNR. The results indicate that all comparisons exhibit high VIF and PSNR values and low SAM and ERGAS values, suggesting that the heatmaps generated by the different CAM methods are highly consistent with each other.

For the VIF index, the highest value for the MSS class is observed between Score-CAM Fast and Grad-CAM (VIF = 0.97), whereas for the MSI_MUT class, the best agreement occurs between Grad-CAM++ and Grad-CAM (VIF = 0.96). These high VIF values confirm that the different CAM methods produce heatmaps that preserve similar visual information.

Regarding the PSNR index, all comparisons show consistently high values, confirming that the generated heatmaps maintain a high level of visual quality among the images. For the MSS class, the highest PSNR value is obtained between Score-CAM Fast and Grad-CAM (PSNR = 32.34), while for the MSI_MUT class, the best agreement is found between Grad-CAM++ and Grad-CAM (PSNR = 34.10).

Conversely, the SAM and ERGAS metrics exhibit low values across all comparisons. For the MSS class, the lowest SAM value is observed between Score-CAM Fast and Grad-CAM (SAM = 0.0499), whereas for the MSI_MUT class, the best agreement occurs between Grad-CAM++ and Grad-CAM (SAM = 0.0581).

Similarly, the lowest ERGAS value for the MSS class is obtained between Score-CAM Fast and Grad-CAM (ERGAS = 1.55), while for the MSI_MUT class, the best agreement is again between Grad-CAM++ and Grad-CAM (ERGAS = 1.73).

Overall, the results summarized in Table 4 confirm that the heatmaps produced by the various CAM methods are visually coherent.

## 4. Discussion

Considering the limitations of traditional molecular methods, several studies have explored deep learning approaches for predicting MSI status directly from digital histopathological images. CNNs can automatically learn and extract complex morphological patterns, thus overcoming the constraints of handcrafted feature-based methods, as shown in this section.

For instance, Chang et al. [19] proposed WiseMSI, a hybrid model combining CNNs with a self-attention mechanism to predict MSI from tiled tumor images, achieving strong performance but offering limited interpretability. Similarly, Wafa and Essa [20] developed various hybrid architectures (CNN-RNN, CNN-LSTM, CNN-GRU) to classify MSI and MSS on TCGA and Kaggle datasets, achieving an accuracy equal to 0.99 while remaining black-box models. Pamuk and Erikçi [21] conducted a comparative analysis on H&E (Hematoxylin and Eosin) images from the Kaggle dataset, with VGG19 reaching the highest performance (accuracy 0.906, precision 0.886, sensitivity 0.931, and AUC 0.906).

Other studies also considered deep learning on H&E slides; for instance, Li et al. [22] developed a model to predict MSI-H in gastroesophageal junction adenocarcinoma, achieving an AUC of 0.933 with an MLP on whole-slide images; Ziegler et al. [23] proposed MiMSI, a deep learning classifier designed for low tumor purity samples, showing sensitivity of 0.895 and AUC of 0.971, outperforming MSISensor; Hezi et al. [24] introduced CIMIL-CRC, integrating pre-trained features and clinical priors for MSI/MSS classification in colorectal cancer, achieving an AUC of 0.92, surpassing patch-based and MIL-only approaches.

Kather et al. [25] used ResNet18 pretrained on ImageNet for tumor and MSI detection in the TCGA and DACHS cohorts, achieving an AUC of 0.84 at the patient level while limiting overfitting through partial weight training. Compared to these results, the networks tested in our study achieved higher performance, with VGG19 (Experiment 8, Table 2) reaching an AUC of 0.976 and integrating Grad-CAM to provide interpretability, further addressing the limitations of black-box models and enhancing transparency of predictions.

In the gastric field, J. Ma et al. [26] combined the VGG16, ResNet50, and MobileNetV2 architectures to improve cancer detection, using LIME to provide interpretable explanations by highlighting the most relevant histological regions. However, techniques like CAM are more effective, as they leverage the internal features of CNNs and generate more precise heatmaps that directly show the areas of the image that influenced the prediction.

### Limitations

Despite the strengths of the proposed method, several limitations should be acknowledged. Although multiple CAM-based methods were employed to visually assess model explainability, we did not perform a detailed analysis of the specific image regions or features highlighted by the heatmaps. Consequently, it remains unclear whether these areas correspond to biologically or pathologically meaningful structures. Future work should aim to validate the interpretability results with the involvement of an experienced pathologist, who could provide expert evaluation of the biological and morphological relevance of the highlighted regions.

## 5. Conclusions

In this paper, we introduced a deep learning-based method for MSI detection in gastrointestinal cancer histology, integrating predictive accuracy with explainability and stability of explanations across different CAM methods. Among the evaluated models, VGG16 and VGG19 networks obtained the best overall performance, achieving interesting accuracy, precision, recall, and AUC scores. As a matter of fact, the experimental evaluation on the TCGA dataset demonstrated that VGG16 and VGG19 achieved the highest performance, with accuracy exceeding 0.92 and AUC values up to 0.976. The adoption of Grad-CAM, Grad-CAM++, and Score-CAM Fast provided explainable heatmaps that consistently highlighted diagnostically relevant tissue regions. Furthermore, the proposed SSIM-based metric demonstrated strong agreement among the CAM methods, reinforcing the stability of generated explanations across different CAM methods. As future work, we aim to incorporate additional explainability methods beyond CAMs, to provide more methods for the evaluation of the prediction of the proposed model.

## Figures and Tables

**Table 1:** The hyperparameters and training times used in the experiments.

Exp	Model	Epochs	Batch Size	Image Size	Learning Rate	Training Time
1	MobileNet	10	16	224 × 3	$1\times 10^{-5}$	2 h 57 m
2	MobileNet	30	32	120 × 3	$1\times 10^{-5}$	3 h 14 m
3	Inception	10	32	120 × 3	$1\times 10^{-5}$	2 h 40 m
4	Inception	6	16	224 × 3	$1\times 10^{-5}$	3 h 52 m
5	VGG16	6	16	224 × 3	$1\times 10^{-5}$	2 h 55 m
6	VGG16	12	16	224 × 3	$1\times 10^{-5}$	5 h 36 m
7	VGG16	12	16	120 × 3	$1\times 10^{-5}$	2 h 09 m
8	VGG19	11	16	224 × 3	$1\times 10^{-5}$	5 h 47 m
9	VGG19	6	16	224 × 3	$1\times 10^{-5}$	3 h 09 m
10	VGG19	12	32	120 × 3	$1\times 10^{-5}$	1 h 56 m
11	VGG19	15	16	224 × 3	$1\times 10^{-5}$	8 h 23 m
12	ViT	10	16	224 × 3	$1\times 10^{-5}$	6 h 57 m

**Table 2:** Model performance across different experiments is reported using Loss, Accuracy, Precision, Recall, F-Measure, and AUC, providing a detailed comparison of the results.

Exp	Model	Loss	Accuracy	Precision	Recall	F-Measure	AUC
1	MobileNet	0.5242	0.8267	0.8267	0.8267	0.8267	0.9060
2	MobileNet	1.1026	0.5722	0.5722	0.5722	0.5722	0.5985
3	Inception	0.4973	0.8055	0.8055	0.8055	0.8055	0.8496
4	Inception	0.4041	0.8827	0.8827	0.8827	0.8827	0.9255
5	VGG16	0.2465	0.9036	0.9036	0.9036	0.9036	0.9670
6	VGG16	0.2685	0.9260	0.9260	0.9260	0.9260	0.9730
7	VGG16	0.4525	0.8739	0.8739	0.8739	0.8739	0.9420
8	VGG19	0.2064	0.9178	0.9178	0.9178	0.9178	0.9760
9	VGG19	0.2850	0.8781	0.8781	0.8781	0.8781	0.9513
10	VGG19	0.5913	0.8517	0.8517	0.8517	0.8517	0.9204
11	VGG19	0.7269	0.4295	0.4295	0.4295	0.4295	0.4055
12	ViT	0.9226	0.6812	0.6915	0.6668	0.6819	0.6972

**Table 3:** The results related to the SSIM metric computation.

CAMs	MSS	MSI_MUT
Grad-CAM++ vs. Grad-CAM	0.6013	0.8447
Score-CAM Fast vs. Grad-CAM	0.9347	0.6243
Grad-CAM++ vs. Score-CAM Fast	0.5764	0.6084

**Table 4:** Comparison of VIF, SAM, ERGAS, and PSNR metric results for different CAM method pairs.

CAMs	VIF		SAM	
	MSS	MSI_MUT	MSS	MSI_MUT
Grad-CAM++ vs. Grad-CAM	0.9188	0.9686	0.1549	0.0581
Score-CAM Fast vs. Grad-CAM	0.9722	0.9342	0.0499	0.1385
Grad-CAM++ vs. Score-CAM Fast	0.9261	0.9314	0.1604	0.1374
**CAMs**	**ERGAS**		**PSNR**	
	**MSS**	**MSI_MUT**	**MSS**	**MSI_MUT**
Grad-CAM++ vs. Grad-CAM	4.612	1.733	23.222	34.099
Score-CAM Fast vs. Grad-CAM	1.554	4.001	32.338	25.400
Grad-CAM++ vs. Score-CAM Fast	4.791	3.985	22.797	26.234

## Data Availability

The data presented in this study are openly available in the Kaggle web repository at following url: https://www.kaggle.com/datasets/joangibert/tcga_coad_msi_mss_jpg, accessed on 6 October 2025.
